# Complete Genome Sequence of *Listeria monocytogenes* Strain DPC6895, a Serotype 1/2b Isolate from Bovine Raw Milk

**DOI:** 10.1128/genomeA.00629-15

**Published:** 2015-06-11

**Authors:** Aidan Casey, Olivia McAuliffe, Aidan Coffey, Karen Hunt, Seamus Fanning, Edward Fox, Kieran Jordan

**Affiliations:** a Teagasc Food Research Centre, Moorepark, Fermoy, Co. Cork, Ireland; b Department of Biological Sciences, Cork Institute of Technology, Bishopstown, Cork, Ireland; c School of Public Health, University College Dublin, Belfield, Dublin, Ireland; d CSIRO Animal Food and Health Sciences, Victoria, Australia

## Abstract

*Listeria monocytogenes* is a foodborne pathogen and is the causative agent of listeriosis among humans and animals. The draft genome sequence of *L. monocytogenes* DPC6895, a serotype 1/2b strain isolated from the raw milk of a cow with subclinical bovine mastitis, is reported.

## GENOME ANNOUNCEMENT

*Listeria monocytogenes* is a Gram-positive facultative anaerobe responsible for the bacterial infection listeriosis. While the disease itself has a low incidence, it is associated with a particularly high mortality rate of 20 to 30% in humans (1). Approximately 95% of all listeriosis cases arise following consumption of food products that are contaminated with *L. monocytogenes* strains of the 1/2a, 1/2b, 1/2c, or 4b serotype (2). In animals such as sheep, goats, and cattle, ingestion of *L. monocytogenes* can result in a wide range of disease manifestations including mastitis, encephalitis, and perinatal mortality, though most infections are subclinical and generally proceed undiagnosed (3). *L. monocytogenes* DPC6895 is a serotype 1/2b strain that was isolated from bovine raw milk during routine sampling on an Irish dairy farm (4). Direct shedding of the bacterium in the raw milk from a cow with subclinical bovine mastitis was identified as the source. It was determined that *L. monocytogenes* DPC6895 persisted in the cow’s udder over a 6-month period, despite intervention with a number of mammary-applied antibiotic treatments.

Bacterial DNA from strain DPC6895 was prepared using the Nextera XT library preparation kit (Illumina, USA), and paired-end (250 bp) sequencing was performed on the Illumina MiSeq platform (Illumina, USA). Quality filtering, adapter clipping, and trimming of the resulting reads were carried out with Trimmomatic, version 0.22 (5). Assembly was then performed using the SPAdes genome assembler tool, version 2.5.1 (6). Open reading frames (ORFs) were predicted using Glimmer v3.02 (7) and RAST (8). The genome was annotated using the RAST server, with subsequent annotations verified and manually curated using BLASTp (9) and Artemis (10).

Sequence assembly yielded a 2,919,539 bp draft genome with >40× average coverage (G+C content of 37.8%), consisting of 9 nonoverlapping contigs, with a contig *N*_50_ size of 347,066 bp and a maximum contig size of 1,159,916 bp. Whole-genome annotation determined that strain DPC6895 contained a total of 2,874 protein coding genes and 53 tRNAs. Multidrug resistance protein clusters, as well as several gene products that putatively function in enhancing resistance of the bacterium to antibiotics were identified. These included fosfomycin (TZ05_1701c), β-lactams (TZ05_0946), vancomycin (TZ05_1643c, TZ05_1695c), tetracycline (TZ05_0838c), lincomycin (TZ05_0528), and quinolone (TZ05_2836c). Additionally, a number of antimicrobial resistance genes were identified, including *telA* (TZ05_1962), which contributes to the resistance of *L. monocytogenes* to the bacteriocin nisin (11), as well as the quaternary ammonium compound resistance transporter proteins SugE-1 and SugE-2 (TZ05_0852 and TZ05_0853). These findings are consistent with previous research which indicated a high prevalence for antibiotic and antimicrobial resistance genes among *L. monocytogenes* dairy isolates (12), and further investigation may provide an insight into how this organism is able to survive and persist in a number of unfavorable environmental conditions.

### Nucleotide sequence accession numbers.

This whole-genome shotgun project for *L. monocytogenes* DPC6895 has been deposited at DDBJ/EMBL/GenBank under the accession no. LABG00000000. The version described in this paper is version LABG01000000.
